# *Smad4* Heterozygous Knockout Effect on Pancreatic and Body Weight in F1 Population Using Collaborative Cross Lines

**DOI:** 10.3390/biology13110918

**Published:** 2024-11-12

**Authors:** Osayd Zohud, Iqbal M. Lone, Kareem Midlej, Aysar Nashef, Fuad A. Iraqi

**Affiliations:** 1 Department of Clinical Microbiology and Immunology, Faculty of Medicine and Health Sciences, Tel-Aviv University, Tel Aviv 6997801, Israel; osaydzohud@mail.tau.ac.il (O.Z.); iqbalzoo84@gmail.com (I.M.L.); kareemmidlej@mail.tau.ac.il (K.M.); 2 Department of Oral and Maxillofacial Surgery, Baruch Padeh Medical Center, Poriya 1528001, Israel; dr.aysarn@gmail.com; 3 Azrieli Faculty of Medicine, Bar-Ilan University, Ramat Gan 5290002, Israel; 4 Department of Oral and Maxillofacial Surgery, Meir Medical Center, Kfar Saba Affiliated to the Faculty of Medicine and Health Sciences, Tel-Aviv University, Tel Aviv 6997801, Israel

**Keywords:** *Smad4*, pancreatic weight, genetic background, sex differences, collaborative cross, F1 mice, genetic modifiers, tumor suppressor gene

## Abstract

*Smad4*, a critical tumor suppressor gene, plays a significant role in pancreatic biology and tumorigenesis. Genetic background and sex are known to influence phenotypic outcomes, but their impact on pancreatic weight in *Smad4*-deficient mice remains unclear. This study investigates the impact of *Smad4* deficiency on pancreatic weight in first-generation (F1) mice from diverse collaborative cross (CC) lines, focusing on the influence of genetic background and sex. F1 mice were generated by crossbreeding female CC mice with C57BL/6J-Smad^4tm1Mak^ males. Genotyping confirmed the presence of *Smad4* knockout alleles. Mice were housed under standard conditions, euthanized at 80 weeks, and their pancreatic weights were measured, adjusted for body weight, and analyzed for effects of Smad4 deficiency, sex, and genetic background. The overall population of F1 mice showed a slight but non-significant increase in adjusted pancreatic weights in heterozygous knockout mice compared to wild-type mice. Sex-specific analysis revealed no significant difference in males but a significant increase in adjusted pancreatic weights in heterozygous knockout females. Genetic background analysis showed that lines CC018 and CC025 substantially increased adjusted pancreatic weights in heterozygous knockout mice. In contrast, other lines showed no significant difference or varied non-significant changes. The interplay between genetic background and sex further influenced these outcomes. *Smad4* deficiency affects pancreatic weight in a manner significantly modulated by genetic background and sex. This study highlights the necessity of considering these factors in genetic research and therapeutic development, demonstrating the value of the collaborative cross mouse population in dissecting complex genetic interactions.

## Simple Summary

This study investigated how a deficiency in the *Smad4* gene affects pancreatic weight in mice with different genetic backgrounds. *Smad4* is important in preventing tumor growth and plays a key role in pancreas health. Researchers focused on F1 mice, which were bred from diverse genetic lines, to explore how genetic variations and sex influence the impact of *Smad4* deficiency. This study explores how Smad4 deficiency affects pancreatic weight across genetic backgrounds and sexes, focusing on physiological impacts beyond its known role in tumor suppression. Overall, *Smad4*-deficient mice showed a slight increase in pancreatic weight compared to normal mice, but this was not statistically significant. Interestingly, female mice had a notable increase in pancreatic weight when they lacked one copy of *Smad4*, while males did not. Additionally, some lines showed an increase in pancreatic weight, while others did not. These findings suggest that both genetic background and sex significantly influence the effects of *Smad4* deficiency on the pancreas. This research highlights the importance of considering individual genetic differences and sex in developing treatments for pancreatic diseases. Understanding how *Smad4* functions could lead to more personalized approaches for managing conditions like pancreatic cancer.

## 1. Introduction

Pancreatic weight measurement is a vital tool in understanding pancreatic health and disease progression, particularly in mouse models. By quantifying pancreatic weight and calculating the pancreas wet weight-to-body weight ratio, researchers can assess changes in organ size, track growth patterns, and evaluate the impact of interventions on pancreatic morphology. These measurements provide insights into the relationship between pancreatic size, metabolic parameters, and underlying pathologies such as inflammation, edema, hypertrophy, or hyperplasia. Studies have shown that these parameters are crucial for monitoring disease development and treatment responses, particularly in conditions like diabetes, pancreatitis, and pancreatic cancer. Thus, incorporating pancreatic weight measurements enhances the reliability of research findings and advances our understanding of pancreatic biology and disease mechanisms [1,2,3].

*Smad4*, also known as *DPC4*, is a critical tumor suppressor gene implicated in various cancers, including hepatocellular carcinoma, breast invasive ductal carcinoma, pancreatic cancer, colorectal cancer, and prostate cancer [4,5,6,7,8]. Loss or reduced expression of *Smad4* is strongly associated with tumor progression, metastasis, and poor prognosis across these cancers [4,6,8]. The importance of understanding *Smad4*’s structure, function, and regulatory mechanisms is emphasized, given its potential prognostic value in pancreatic cancer. Studies suggest that analyzing *Smad4* protein expression in pancreatic cancer cell lines can reveal insights into the molecular mechanisms driving pancreatic cancer, offering avenues for novel therapeutic strategies targeting *Smad4*-deficient tumors, to improve patient outcomes. Further research is needed to elucidate *Smad4*’s role in pancreatic cancer progression and to explore innovative treatments based on this understanding [9]. *Smad4* plays a key role in the TGF-β (transforming growth factor-beta) signaling pathway by forming complexes with other Smad proteins to regulate gene transcription, a process crucial for controlling cellular proliferation and differentiation [5,7]. Research has demonstrated that mutations or decreased expression of *Smad4* correlate with advanced disease stages, lymph node metastasis, and reduced survival rates in cancer patients, highlighting its potential as a target for therapeutic intervention and prognostic assessment in oncology [8].

The TGF-β/Smad4 signaling pathway is further recognized for its regulation of key biological processes, including epithelial–mesenchymal transition, DNA damage response, and microRNA regulation, with *Smad4* acting as a crucial tumor suppressor. Interactions between the TGF-β/Smad4 pathway and other signaling cascades like MAP kinase, PI3K/Akt/mTOR, and WNT/β-catenin are particularly significant in tumor formation and progression, pointing to *Smad4* as a potential target for cancer therapies [10]. Specifically, in pancreatic cancer, *Smad4* serves as a tumor suppressor by inhibiting epithelial cell proliferation, and its loss can lead to tumor-promoting effects, illustrating the dual role of TGF-β in this malignancy [7].

Genetic background plays a pivotal role in shaping phenotypic variation and responses to genetic perturbations, making it essential to understand how genetic modifiers influence the outcomes gene mutations [11]. This study investigates the impact of genetic background on pancreatic phenotypes in *Smad4* knockout F1 mice derived from diverse collaborative cross (CC) lines. By generating F1 mice carrying both CC and *Smad4* knockout alleles and measuring pancreatic and body weights across multiple CC lines, we assess how genetic variation affects pancreatic development and growth in the context of *Smad4* deficiency.

The collaborative cross (CC) is a genetically defined recombinant mouse panel derived from the systematic interbreeding of eight founder strains, including five classically used inbred strains (A/J, C57BL/6J, 129S1/SvlmJ, NOD/ShiLtJ, NZO/HILtJ) and three wild-derived strains (CAST/EiJ, PWK/PhJ, WSB/EiJ) [12]. This breeding strategy resulted in a panel where each new CC strain is more than 90% homozygous, yet retains extensive genetic variation uniformly distributed across the genome. The CC population captures a broad spectrum of allelic diversity, representing over 50 million sequence variants across more than 50 strains. This genetic complexity mirrors that found in natural mouse populations and offers a significant advantage over traditional laboratory strains with limited genetic diversity [13]. By providing a diverse genetic platform, the CC strains facilitate the dissection of the genetic architecture underlying complex traits, enabling researchers to develop new disease models and explore phenotype diversity. The CC mice are widely used across various research fields, including immunology, infectious diseases, oncology, neurobiology, and toxicology, allowing for detailed studies and discovery of new genetic and phenotypic insights [14].

The CC population also facilitates investigating gene–gene interactions, or epistasis, which are crucial in determining phenotypic outcomes [14]. Through systematic crossing and intercrossing of CC lines, researchers can explore how multiple genetic loci interact to influence phenotypic traits, leading to the identification of genetic modifiers that modulate the effects of primary genetic perturbations [15]. This approach is particularly valuable for understanding the genetic determinants of pancreatic phenotypes, given the pancreas’ role in metabolic homeostasis, digestion, and hormone regulation. Dysregulation of pancreatic development and function is linked to various diseases, including diabetes, pancreatic cancer, and exocrine insufficiency.

Our study leverages the genetic diversity of the CC mouse population to identify genetic modifiers that influence the phenotypic consequences of *Smad4* knockout, a key regulator of cellular signaling pathways involved in pancreatic development and tumorigenesis. By uncovering the molecular pathways underlying pancreatic phenotypes in the context of genetic variation, we aim to enhance our understanding of pancreatic biology and contribute to the development of targeted therapies and precision medicine approaches for pancreatic diseases.

## 2. Materials and Methods

### 2.1. Ethics and Animal Welfare Considerations

This research adhered to the national guidelines for the ethical treatment of laboratory animals. The study’s protocol received approval from the Institutional Animal Care and Use Committee (IACUC) of Tel Aviv University in 2019, under the authorization number (01-19-044). Daily health monitoring of the mice was conducted, with specific criteria set for humane euthanasia based on weight loss or observed distress, in consultation with the facility’s veterinary staff.

### 2.2. Crossbreeding to Generate F1 Offspring

The collaborative cross (CC) mouse strains were developed and propagated at Tel-Aviv University’s animal facility under standard conditions through about 20 generations of inbreeding, using previously described methodologies [16]. Seven CC lines were used in this experiment. The C57BL/6 J-Smad4^tm1Mak^ strain was sourced from the Jackson Laboratory (Bar Harbor, ME, USA). Crosses between female mice from available CC strains and C57BL/6 J-Smad4^tm1Mak^ males resulted in F1 offspring. F1 mice in this experiment represent a blend of genetic backgrounds from the CC strains and the C57BL/6 J-Smad4^tm1Mak^ strain, offering a rich source of genetic and phenotypic diversity for further study. Their unique genetic makeup and ability to produce intermediate phenotypes make them valuable for investigating the effects of specific genetic alterations on various traits. A total of 122 pancreas samples were collected and weighed in this study, comprising 64 males (33 heterozygous knockouts [KO] and 31 wild-types [WT]) and 58 females (27 heterozygous knockouts [KO] and 31 wild-types [WT]), with the mice distributed across seven collaborative cross lines. Full details of the number of F1 mice across lines for inclusion in subsequent experiments per line are presented in Table 1.

### 2.3. Animal Housing and Nutritional Care

The mice were accommodated at the Sackler Faculty of Medicine’s animal facility, Tel-Aviv University, under conditions approved by the university’s Animal Use and Care Committee (01-19-044). They were kept in cages with hardwood chip bedding, separated by sex and CC lineage, under a consistent 12 h light/dark cycle, at a room temperature of 22 °C. From weaning at three weeks until the end of the study at 80 weeks, they had unrestricted access to water and a standard rodent diet (TD.2018SC, Teklad Global, Harlan Inc., Madison, WI, USA).

### 2.4. Extraction of Genomic DNA

For genomic DNA extraction, the NaOH method was employed, as referenced in [17]. Tail samples measuring 3–4 mm were collected into Eppendorf tubes, to which a mixture of 75 µL of 25 mM NaOH and 0.2 mM EDTA was added. These samples were then heated at 98 °C for 1 h in a thermocycler, cooled to 15 °C, and neutralized with 75 µL of 40mM Tris HCl (pH 5.5) post-heating. Centrifugation at 4000 rpm for 3 min helped clarify the samples, which were then ready for PCR genotyping.

### 2.5. F1 Mouse PCR Genotyping

Specific three-set primer pairs were used for PCR-based genotyping:

- Primer 30403 (5′-TGT AGT TCT GTC TTT CCT TCC TG-3′)
- Primer 30404 (5′-ACT GAC CTT TAT ATA CGC GCT TG-3′)
- Primer oIMR2088 (5′-AGA CTG CCT TGG GAA AAG CG-3′)

Two PCR reactions were set up:

Reaction A targeted a 200 bp segment of the *Smad4* gene’s wild-type allele using primers 30403 and 30404.

Reaction B aimed to amplify a 300 bp fragment indicative of the *Smad4* knockout allele with primers 30404 and oIMR2088.

**Figure 1:**
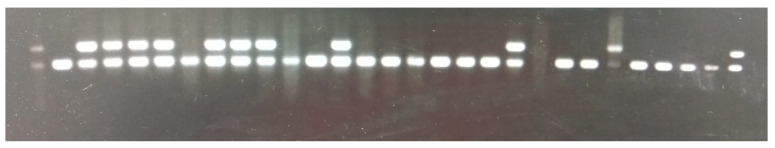
PCR genotyping of mice. Lanes with two bands at 200 base pairs (bp) and 300 bp indicate positive carriers of the *Smad4* mutation, while lanes with only a 200 bp band represent wild-type mice. The first lane shows the 100 bp DNA ladder marker for size reference. The uncropped version is available in Supplementary Figure S1, in the Supplementary Materials.

Both reactions constitute a touchdown phase. Afterward, the PCR resumed with denaturation at 94.0 °C, annealing at 60.0 °C, and extension at 72.0 °C for 30 cycles. Finally, an extension step was conducted at 72.0 °C, followed by a hold step at 10.0 °C, as instructed in the original protocol (https://www.jax.org/Protocol?stockNumber=029250&protocolID=20424) URL (accessed on 1 October 2019). After PCR amplification, the products were analyzed via agarose gel electrophoresis. A 2% agarose gel containing ethidium bromide was prepared, and PCR samples were loaded alongside a 100 bp DNA ladder for size reference. The gel was run in TBE buffer at a voltage of 100 volts for 30 min. Following electrophoresis, the gel was visualized under UV light, revealing distinct bands. The observed bands corresponded to the wild-type allele (200 bp) and the knockout allele (300 bp). Figure 1 shows PCR gel images that illustrate the genotyping of heterozygous *Smad4* KO mice. These images show the distinct PCR bands for wild-type and heterozygous knockout genotypes, providing clarity on the genotyping results and confirming the successful introduction of the heterozygous *Smad4* KO into the CC mouse model.

### 2.6. Tissue Harvesting

At 80 weeks, the mice were humanely euthanized using CO2, and their final body weights were recorded. Body weight changes were calculated using the formula: (final body weight − initial body weight) × 100%/final body weight [18]. The pancreas was extracted, weighed, and the weight was adjusted relative to the body weight using the formula: pancreas weight × 100% body weight [19].

### 2.7. Data Analysis

IBM SPSS Statistics, Version 23.0 (IBM Corp., Armonk, NY, USA, 2015), was used for data analysis. An independent sample *t*-test was conducted to compare adjusted pancreatic weights between knockout (KO) and wild-type (WT) groups. The analysis also included assessing the impact of sex on adjusted pancreatic weights and the variation in adjusted pancreatic weights across different genetic lines to understand line effects.

## 3. Results

### 3.1. The Effect of Smad4 Kock out in the General Population of F1 Mice

**Figure 2:**
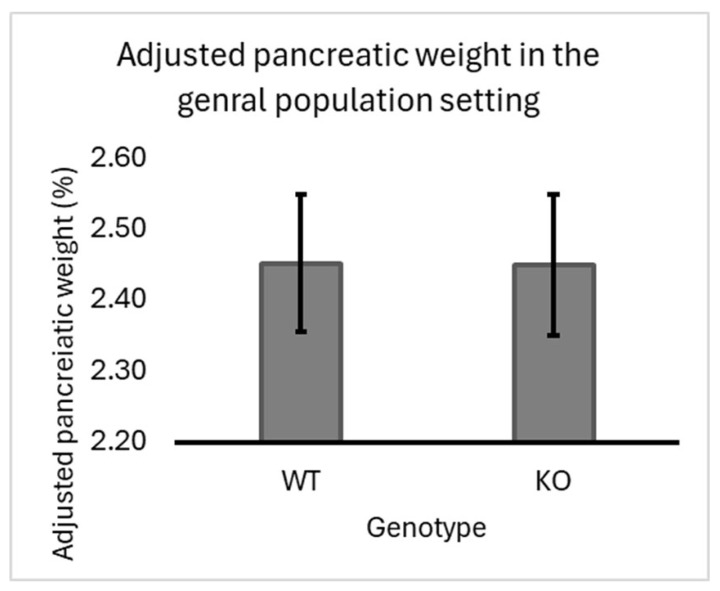
Impact of *Smad4* deficiency on adjusted pancreatic weight in the general population of F1 Mice: The graph represents the adjusted pancreatic weights (pancreas weight as a percentage of body weight) for wild-type (WT) and heterozygous knockout (KO) (*Smad4*+/−) F1 mice across 7 different genetic lines of the collaborative cross (CC) population. The overall trend shows a slight increase in pancreatic weight in the *Smad4*+/− group compared to the WT group, although this increase is not statistically significant (*p >* 0.05). Error bars denote the standard error of the mean (SEM). The lack of substantial difference suggests that *Smad4* deficiency has a modest effect on pancreatic weight in the general population. The statistical significance of differences in the average adjusted pancreatic weights between the two groups was calculated.

Our experiment’s overall population of F1 mice (regardless of sex or line) showed a slight but insignificant increase in pancreatic-adjusted weight in heterozygous knockout mice (2.51%) compared to wild-type (2.47%) seven-line crosses. Data supporting this finding are presented in Figure 2.

### 3.2. Sex Effect

**Figure 3:**
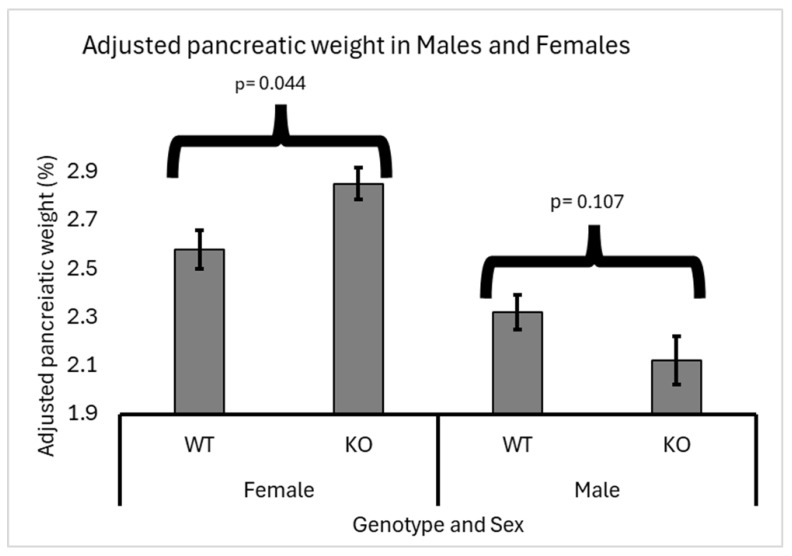
Sex-specific effects of *Smad4* deficiency on adjusted pancreatic weight in F1 mice: The graph illustrates the adjusted pancreatic weights (pancreas weight as a percentage of body weight) for wild-type (WT) and heterozygous knockout (KO) male and female F1 mice. In male mice, there is no significant difference in adjusted pancreatic weights between WT and *Smad4*+/− groups (*p =* 0.061). In contrast, female mice show a significant increase in adjusted pancreatic weights in the *Smad4*+/− group compared to the WT group (*p =* 0.041). Error bars represent the standard error of the mean (SEM). These results highlight a sex-specific response to *Smad4* deficiency, with females exhibiting a more pronounced increase in pancreatic weight than males.

Heterozygous knockout males showed a slight decrease in adjusted pancreatic weights (2.22%) compared to wild-type males (2.37%). Meanwhile, in females, there was a significant increase in adjusted pancreatic weights in heterozygous knockout mice (2.9%) compared to wild-type mice (2.6%). Data supporting these findings are presented in Figure 3. These results suggest a sex-specific effect of heterozygous knockout on pancreatic weight regulation, highlighting the importance of further investigation into the underlying mechanisms driving this observed disparity.

### 3.3. Line Genetic Effect

**Figure 4:**
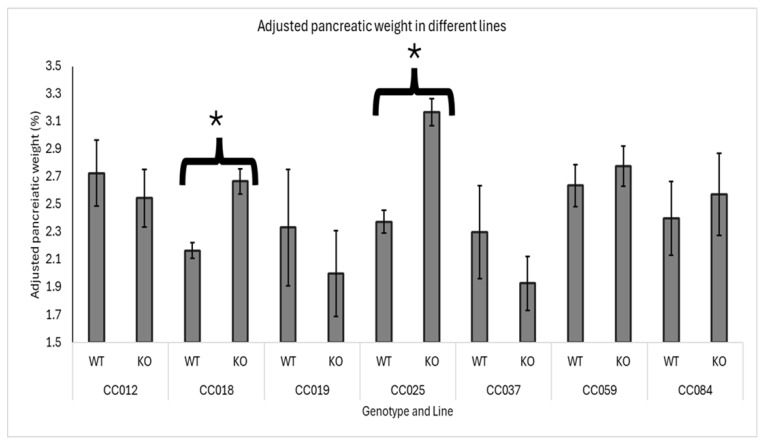
Line-specific effects of *Smad4* deficiency on adjusted pancreatic weight in F1 mice: The graph presents the adjusted pancreatic weights (pancreas weight as a percentage of body weight) for wild-type (WT) and heterozygous knockout (*Smad4*+/−) F1 mice across various collaborative cross (CC) lines. Error bars represent the standard error of the mean (SEM). The statistical significance of differences in the adjusted pancreatic weights between the two groups is presented as follows: (*) indicates a significant difference at *p <* 0.05, CC018: *p =* 0.041 and CC025: *p =* 0.037.

The effect of *Smad4* knockout on adjusted pancreatic weights varied significantly among the different genetic lines of F1 mice. Lines CC018 (WT mean = 2.15%, KO mean = 2.67%) and CC025 (WT mean = 2.36%, KO mean = 3.2%) exhibited a significant increase in adjusted pancreatic weights in heterozygous knockout mice compared to wild-type mice, indicating a notable impact of *Smad4* deficiency in these specific genetic backgrounds. Conversely, lines CC012, CC019, CC037, CC059, and CC084 showed no significant difference in adjusted pancreatic weights between heterozygous knockout and wild-type mice, suggesting that *Smad4* deficiency does not markedly affect pancreatic weight adjustment in these genetic backgrounds. Among these, lines CC012, CC059, and CC084 showed no change in adjusted pancreatic weights, meaning that the pancreatic weight relative to body weight was consistent between heterozygous knockout and wild-type mice. Additionally, lines CC019 and CC037 presented with a non-significant decrease in adjusted pancreatic weights in heterozygous knockout mice compared to wild-type mice, suggesting that the effect of *Smad4* deficiency might be subtle or influenced by other genetic or environmental factors in these lines. Data supporting these findings are presented in Figure 4, which details the specific adjusted pancreatic weights for each genetic line, highlighting the variability in response to *Smad4* knockout across different genetic backgrounds.

### 3.4. Line and Sex Effect

**Figure 5:**
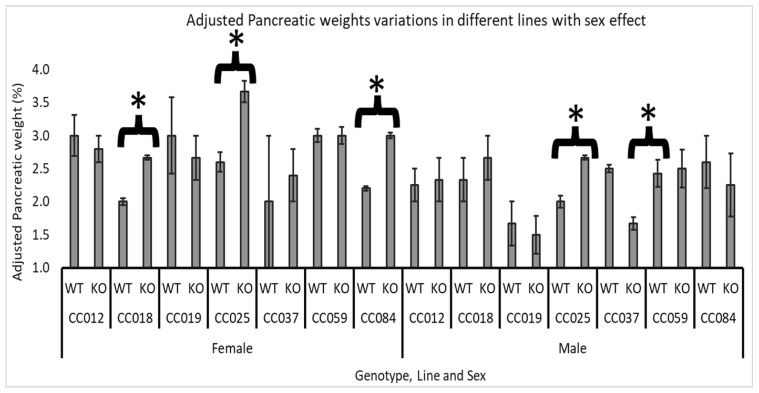
Sex and line-specific effects of *Smad4* deficiency on adjusted pancreatic weight in F1 mice: The graph shows the adjusted pancreatic weights (pancreas weight as a percentage of body weight) for male and female wild-type (WT) and heterozygous knockout (*Smad*4+/−) F1 mice across seven collaborative cross (CC) lines. Error bars represent the standard error of the mean (SEM). The statistical significance of differences in the average adjusted pancreatic weights between the two groups is presented as follows: (*) indicates a significant difference at *p <* 0.05. CC018, female: *p =* 0.016, CC025, female: *p =* 0.04, CC084, female: *p =* 0.007, CC025, male: *p =* 0.016 and CC037, male: *p =* 0.027.

In this section, we compared the impact of *Smad4* deficiency across seven lines (CC012, CC018, CC019, CC025, CC037, CC059, and CC084), analyzing differences in adjusted pancreatic weights between male and female mice. The results of this section are presented in Figure 5.

For female mice, a significant effect of *Smad4* knockout was observed in three lines: CC018 (WT mean = 1.98%, KO mean = 2.67%), CC025 (WT mean = 2.6%, KO mean = 3.67%), and CC084 (WT mean = 2.19%, KO mean = 3.04%). This indicates that *Smad4* deficiency substantially increases adjusted pancreatic weights, specifically in females from these lines, suggesting a heightened sensitivity or a different regulatory mechanism in female mice in response to the knockout.

In male mice, the impact of *Smad4* deficiency was less consistent across the lines. Only line CC025 significantly increased adjusted pancreatic weights (WT mean = 1.98%, KO mean = 2.7%), mirroring the effect seen in females of the same line. Additionally, line CC037 showed a significant decrease in adjusted pancreatic weights in male mice (WT mean = 2.5%, KO mean = 1.6%), contrasting with the lack of substantial change or increase in other lines and sexes. This suggests that the impact of *Smad4* deficiency may not only be sex specific but also vary widely depending on the genetic background of the mice.

### 3.5. Heritability

This study aimed to discover whether adjusted pancreatic weight phenotypic variance has a genetic basis in *Smad4* knockout F1 populations. Table 2 summarizes the heritability (H2) values calculated to answer this question. One-way ANOVA was used to calculate the heritability of sex- and genotype-specific characteristics. The traits calculated are pancreatic-adjusted weights and body weight changes for both sexes and genotypes.

## 4. Discussion

The *Smad4* gene under scrutiny encodes a signal transduction that is essential for both embryonic development and signaling. This gene has been linked to Myhre syndrome, juvenile polyposis syndrome, hereditary hemorrhagic telangiectasia syndrome, and human pancreatic cancer [20,21]. The knockout mice used in this study carry a mutation, where a NEO selection cassette replaces exon 8 and a portion of exon 9 in the *Smad4* gene. While it is known from previous studies that homozygous null mice for this mutation are embryonically lethal and exhibit severe developmental abnormalities [22,23], our research focused exclusively on heterozygous knockout mice. These mice are viable and fertile, allowing us to explore the genetic interactions affecting pancreatic weight in the context of a heterozygous *Smad4* knockout. [24,25].

Our study aimed to investigate the effect of *Smad4* deficiency on pancreatic weight in F1 mice across different genetic backgrounds using the collaborative cross (CC) lines. This study builds upon our previous research with CC and Smad4 knockout models, focusing on how different genetic backgrounds influence phenotypic outcomes. We primarily explored the impact of *Smad4* heterozygosity on body weight across different genetic backgrounds. The study highlighted significant weight variability influenced by genetic diversity in CC mouse lines, identifying specific lines with pronounced susceptibility to weight gain under *Smad4* knockout conditions [26]. Additionally, we have highlighted the role of genetic background on juvenile polyposis syndrome (JPS) development in *Smad4*-deficient mice, revealing line-specific variations in intestinal polyp development [18]. The findings provide insights into how *Smad4* knockout influences pancreatic morphology, revealing significant variability influenced by genetic background and sex. Previous reports suggested *Smad4* loss alone does not affect pancreatic weight, as mice with homozygous deletion of *Smad4* in the pancreas did not show any gross anatomical or physiological abnormalities, maintaining normal pancreatic cytoarchitecture. Pancreatic weight remained unaffected in this context [27,28].

Additionally, another report on transgenic mice expressing dn*Smad4*, a dominant-negative *Smad4* protein, does not show significant changes in pancreatic weight, exocrine, or ductal histology compared to wild-type mice. However, these mice display an age-dependent increase in islet size. The dn*Smad4* transgene expression leads to an expanded population of replicating cells expressing the transgene in the stroma between enlarged islets and pancreatic ducts. This suggests that loss of *Smad4* signaling in the pancreas does not affect overall pancreatic weight but influences islet size and cell proliferation dynamics within the pancreatic tissue [29,30,31,32].

The overall population of F1 mice showed a slight but not statistically significant increase in adjusted pancreatic weights in heterozygous knockout mice compared to wild-type mice. This suggests that *Smad4* deficiency might influence pancreatic weight, but the effect is modest and could be masked by other genetic and environmental factors. Previous studies have established *Smad4*’s role in cellular signaling pathways and its involvement in various cancers, including pancreatic cancer [10,33]. Our findings align with the idea that *Smad4* has a complex role in pancreatic biology, potentially affecting growth and morphology in subtle ways that warrant further investigation.

The impact of *Smad4* knockout on adjusted pancreatic weights varied significantly between male and female mice. Heterozygous knockout males showed a slight decrease in adjusted pancreatic weights compared to wild-type males. However, a significant increase in adjusted pancreatic weights was noted in females. This sex-specific response suggests that female mice might be more sensitive to *Smad4* deficiency, potentially due to differences in hormonal regulation or metabolic processes that influence pancreatic development and function. The sexual dimorphism observed in our study could be crucial for understanding how genetic mutations differentially affect males and females and might help tailor sex-specific therapeutic approaches in diseases related to *Smad4* deficiency.

The response to *Smad4* knockout varied across different CC lines, indicating that genetic background significantly influences the phenotypic outcome, as also revealed by Qahaz et al. [26,34]. Lines CC018 and CC025 showed a significant increase in adjusted pancreatic weights in heterozygous knockout mice compared to wild-type mice. These lines may harbor genetic modifiers interacting with *Smad4*, enhancing its impact on pancreatic weight. Conversely, lines CC012, CC019, CC037, CC059, and CC084 did not show significant differences, suggesting that other compensatory mechanisms might mitigate the effect of *Smad4* deficiency in these genetic backgrounds. The non-significant increases and decreases observed in some lines further highlight the complexity of genetic interactions that regulate pancreatic morphology.

The current research adds a new dimension by investigating both pancreatic weight and sex-specific phenotypic responses in *Smad4*-deficient mice, findings not addressed in our previous publications [18,26]. Unlike our earlier studies, which primarily concentrated on body weight and polyp formation, this study presents a focused exploration of pancreatic morphology and sex-dependent physiological outcomes. Our data reveal that *Smad4* deficiency leads to a distinct increase in pancreatic weight in female mice, particularly within specific CC lines such as CC018 and CC025, demonstrating a unique genetic and sex-based interaction previously unreported. These findings, combined with our past results, underscore the extensive variability in Smad4-related phenotypes across genetically diverse backgrounds, highlighting *Smad4*’s role in mediating phenotypic diversity, particularly under the influence of sex and genetic background, thus advancing our understanding of Smad4’s function in complex trait regulation and disease susceptibility.

The analysis of sex effects within specific lines revealed additional complexity. In female mice, a significant impact of *Smad4* knockout was observed in lines CC018, CC025, and CC084, whereas in male mice, significant changes were noted only in lines CC025 (increase) and CC037 (decrease). This highlights the importance of considering genetic background and sex when studying the effects of genetic mutations. The significant reduction in adjusted pancreatic weights in CC037 males, contrasting with the effects in females, underscores the intricate interplay between genetic, hormonal, and environmental factors in shaping phenotypic outcomes. These findings suggest that therapeutic strategies for conditions involving *Smad4* might need to be personalized based on both genetic background and sex.

Recent advancements in genetic modifications have provided new insights into pancreatic biology, particularly in understanding the genetic mechanisms that influence tumor progression and therapeutic responses. Studies on gene drives, including CRISPR-Cas systems, have demonstrated the potential to modify genetic traits in various organisms rapidly [35]. These techniques, while primarily applied in ecological studies, also highlight the broader implications of precise genetic interventions for disease control, including potential applications in pancreatic cancer research. Furthermore, the investigation of the tumor microenvironment (TME) is crucial in understanding pancreatic cancer’s complexity [36]. For instance, platelets, beyond their traditional role in hemostasis, have emerged as critical players within the TME, influencing tumor progression and serving as vehicles for targeted drug delivery [37]. In pancreatic cancer specifically, patient-derived models such as organoids and xenografts have allowed for a more nuanced understanding of the tumor’s heterogeneity, offering insights into how genetic mutations, such as those in the *Smad4* gene, contribute to tumor growth and resistance to therapy. These diverse research approaches underscore the critical role of genetic and cellular studies in advancing our understanding of pancreatic cancer and developing targeted therapies that address the genetic complexity of this disease.

These findings on *Smad4* deficiency provide valuable insights into the biological mechanisms that govern pancreatic growth and development. The observed effects of *Smad4* loss on pancreatic weight, influenced by genetic background and sex, underscore *Smad4*’s regulatory role in pancreatic physiology and its potential impact on susceptibility to conditions like pancreatic cancer. This study suggests that *Smad4* may interact with genetic and hormonal factors that modulate pancreatic cell proliferation and growth pathways. Clinically, understanding these interactions could lead to novel therapeutic strategies that leverage *Smad4* modulation to control pancreatic growth or inhibit tumorigenesis. Additionally, these findings offer potential applications in developing disease models that incorporate genetic variability and sex-specific factors, facilitating more personalized approaches to studying pancreatic diseases. Expanding on these results in future research could ultimately inform both therapeutic development and the design of genetically diverse animal models for better prediction of treatment outcomes.

While this study provides initial insights into the impact of *Smad4* deficiency on pancreatic weight across various genetic backgrounds and between sexes, further investigation into the underlying mechanisms would provide a more complete understanding of *Smad4*’s role in pancreatic function. Future studies could benefit from evaluating additional parameters, such as pancreatic volume, cell density, insulin secretion, and protein expression levels. These measurements would allow for a more comprehensive assessment of how *Smad4* loss influences both pancreatic structure and function. Moreover, exploring whether genetic background correlates with specific metabolic or hormonal profiles could elucidate the genetic modifiers and molecular pathways involved in the observed variability. Together, these additional analyses would deepen our understanding of the physiological and functional consequences of *Smad4* deficiency, paving the way for potential therapeutic strategies tailored to genetic and sex-specific differences in pancreatic disease susceptibility.

## 5. Conclusions

Our study underscores the importance of genetic background and sex differences in the phenotypic effects of *Smad4* gene knockouts. Using the diverse CC mouse population, we revealed significant variability in pancreatic weight, driven by genetic modifiers and sex-specific responses. These findings highlight the need for further research to identify these modifiers and explore hormonal and gene expression differences, which could inform the development of targeted therapies. Ultimately, considering genetic diversity and sex in research will enhance our understanding of *Smad4*’s role in pancreatic health and lead to more personalized treatments.

## Figures and Tables

**Table 1:** Summarizes the sample size of male and female mice from the seven collaborative crosslines with different genetic backgrounds. (WT) stands for mice with two copies of wild-type *Smad4* gene, while (KO) stands for heterozygous knockout (*Smad4*+/−) F1 mice.

		Line						
		CC012	CC018	CC019	CC025	CC037	CC059	CC084
Female	WT	7	3	3	5	4	4	5
	KO	5	3	3	3	5	5	3
Male	WT	4	3	3	3	6	7	5
	KO	6	3	4	3	9	4	4

**Table 2:** Results of calculating heritability (H2) and genetic variance (VG), and coefficient of genetic variation (CVg) values. Heritability was calculated using one-way ANOVA for the traits in our study, which were calculated separately by sex and genotype.

Sex	Genotype	Trait	VG	H2	CVg
Female	WT	Adjusted Pan Weight %	0.284903	0.477302	0.214363
Female	WT	Delta_BW	14.5507	0.153954	0.062006
Female	KO	Adjusted Pan Weight %	0.257129	0.603407	0.201222
Female	KO	Delta_BW	4.166905	0.036585	0.032808
Male	WT	Adjusted Pan Weight %	0.536923	0.456597	0.319978
Male	WT	Delta_BW	6.331883	0.04312	0.04313
Male	KO	Adjusted Pan Weight %	0.243643	0.412505	0.196654
Male	KO	Delta_BW	4.073576	0.030492	0.03395

## Data Availability

Data are contained within the article.

## Supplementary Materials

The following supporting information can be downloaded at: https://www.mdpi.com/article/10.3390/biology13110918/s1, Figure S1. Uncropped gel image of PCR genotyping for heterozygous Smad4 knockout (KO) mice, displaying all lanes and bands to ensure complete data transparency. The gel includes both 200 bp and 300 bp bands, along with the 100 bp DNA ladder in the first lane, used for size reference.
