# Supplementary material for: Smad4 Heterozygous Knockout Effect on Pancreatic and Body Weight in F1 Population Using Collaborative Cross Lines
Source: Biology (Basel). 2024 Nov 12;13(11):918. doi: 10.3390/biology13110918 (PMC11592182; doi:10.3390/biology13110918)

Supplementary Figure S1. Uncropped gel image of PCR genotyping for heterozygous *Smad4* knockout (KO) mice, displaying all lanes and bands to ensure complete data transparency. The gel includes both 200 bp and 300 bp bands, along with the 100 bp DNA ladder in the first lane, used for size reference.

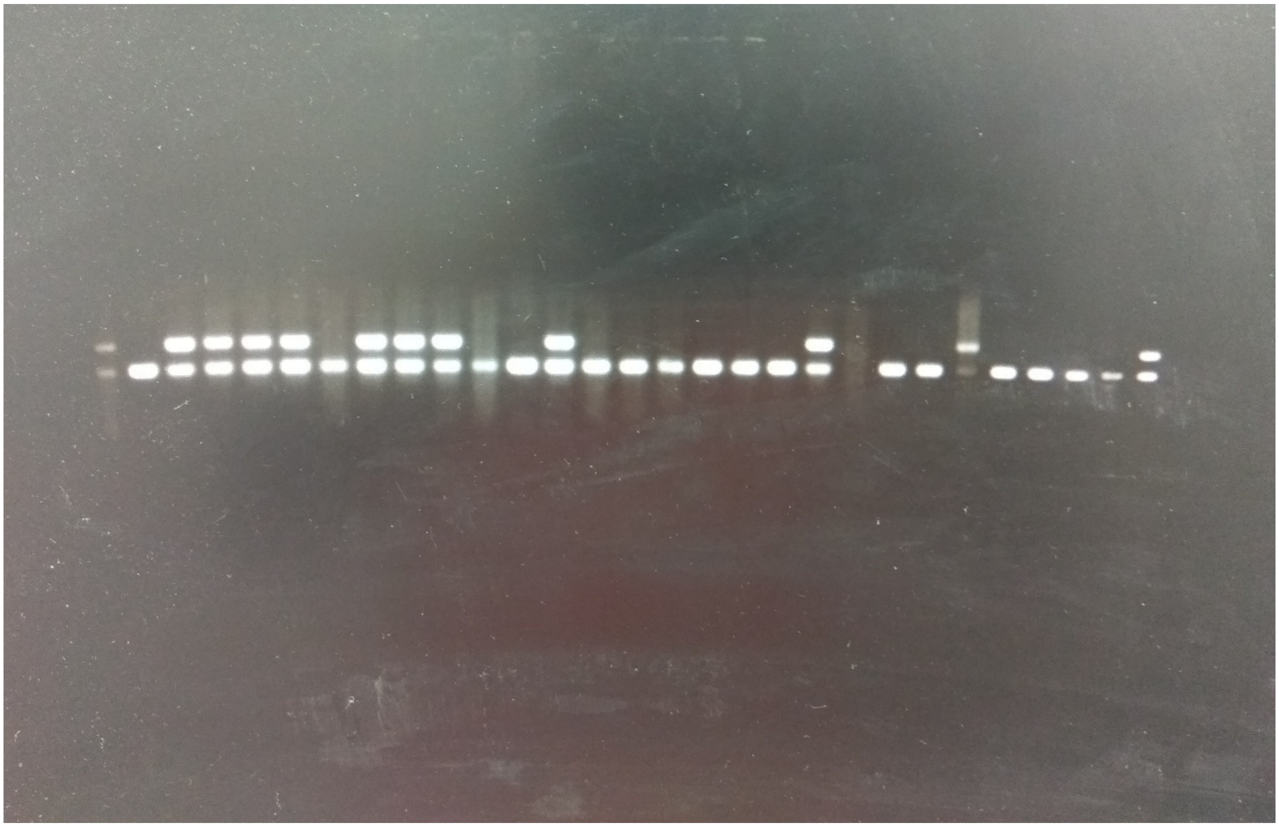

Supplement: Supplementary file 1 [file biology-13-00918-s001.zip › biology-3275832-supplementary.pdf]
